# Krill Oil Alleviated Methamphetamine-Induced Memory Impairment *via* the MAPK Signaling Pathway and Dopaminergic Synapse Pathway

**DOI:** 10.3389/fphar.2021.756822

**Published:** 2021-10-29

**Authors:** Qin Ru, Xiang Tian, Qi Xiong, Congyue Xu, Lin Chen, Yuxiang Wu

**Affiliations:** ^1^ Wuhan Institutes of Biomedical Sciences, School of Medicine, Jianghan University, Wuhan, China; ^2^ Department of Health and Physical Education, Jianghan University, Wuhan, China

**Keywords:** krill oil, methamphetamine, memory deficits, network pharmacology, hippocampus

## Abstract

Methamphetamine (METH) abuse exerts severe harmful effects in multiple organs, especially the brain, and can induce cognitive dysfunction and memory deficits in humans. Krill oil is rich in polyunsaturated fatty acids, while its effect on METH-induced cognitive impairment and mental disorders, and the underlying mechanism remain unknown. The aim of the present study was to investigate the protective effect of krill oil on METH-induced memory deficits and to explore the molecular mechanisms by using an integrated strategy of bioinformatics analysis and experimental verification. METH-exposed mice were treated with or without krill oil. Learning and memory functions were evaluated by the Morris water maze. The drug–component–target network was constructed in combination with network pharmacology. The predicted hub genes and pathways were validated by the Western blot technique. With krill oil treatment, memory impairment induced by METH was significantly improved. 210 predicted targets constituted the drug–compound–target network by network pharmacology analysis. 20 hub genes such as DRD2, MAPK3, CREB, BDNF, and caspase-3 were filtered out as the underlying mechanisms of krill oil on improving memory deficits induced by METH. The KEGG pathway and GO enrichment analyses showed that the MAPK signaling pathway, cAMP signaling pathway, and dopaminergic synapse pathway were involved in the neuroprotective effects of krill oil. In the hippocampus, DRD2, cleaved caspase-3, and γ-H2AX expression levels were significantly increased in the METH group but decreased in the krill oil–treated group. Meanwhile, krill oil enhanced the expressions of p-PKA, p-ERK1/2, and p-CREB. Our findings suggested that krill oil improved METH-induced memory deficits, and this effect may occur *via* the MAPK signaling pathway and dopaminergic synapse pathways. The combination of network pharmacology approaches with experimental validation may offer a useful tool to characterize the molecular mechanism of multicomponent complexes.

## Introduction

As one of the most commonly abused psychostimulants in the world (Siefried et al., 2020), methamphetamine (METH) abuse results in various severe complications systemically affecting multiple organs, especially the brain (Casaletto et al., 2015; Huang et al., 2019). METH addiction can induce neurodegenerative changes in the hippocampus and frontal cortex, which are all related to long-term cognitive dysfunction and memory deficits in humans (Casaletto et al., 2015; Chen et al., 2015; Wang et al., 2018). Long-term METH addiction reduces the abusers’ awareness of memory impairment, and overestimation of memory further exacerbates their executive dysfunction (Casaletto et al., 2015). It is important to note that neurocognitive deficits did not just occur in people who are currently abusing METH but has also been found in those who have stopped taking METH for an extended period of time (Cherner et al., 2010; Silva et al., 2014). In line with these clinical investigations, several studies in animal models also have documented that repeated METH administration could essentially affect different brain areas including the frontal cortex and hippocampus, which are all associated with cognitive and memory function (Fan et al., 2020; Golsorkhdan et al., 2020; Lwin et al., 2020; Veschsanit et al., 2021). However, the potential mechanism of cognitive dysfunction induced by METH is unclear. A deep understanding of its mechanism may provide more valuable ideas for the treatment of METH addiction and its induced mental disorders.

Krill oil is extracted from the Antarctic microcrustacean *Euphausia superba*, and is a rich source of astaxanthin, and (n-3)/polyunsaturated fatty acids (PUFAs), including eicosapentaenoic acid (EPA) and docosahexaenoic acid (DHA) (Alvarez-Ricartes et al., 2018). Due to the high presence of astaxanthin, EPA, and DHA, krill oil has been reported to have positive effects on cardiovascular disease, insulin resistance, lipid and glucose metabolism, and neurocognitive impairment in various animal experiments (Cheong et al., 2017; Sun et al., 2017; Tome-Carneiro et al., 2018). As a food supplement, krill oil has great bioavailability (Sun et al., 2017) and has become popular with some pilot trials and randomized controlled trials indicating healthy benefits. For instance, krill oil could reduce the plasma triacylglycerol level and improves the related lipoprotein particle concentration, fatty acid composition, and redox status in healthy young adults (Berge et al., 2015). Krill oil could modestly improve cardiovascular risk in patients with type 2 diabetes, and krill oil supplementation may lead to a small but significant increase in the mean omega-3 index (Lobraico et al., 2015; van der Wurff et al., 2019; van der Wurff et al., 2020). Krill oil could also activate cognitive function in the elderly and is more effective than sardine oil in the working memory task (Konagai et al., 2013). However, despite the several studies conducted to show the beneficial effects of krill oil on neurocognitive function, the precise mechanism of krill oil on neurocognitive dysfunction in the central nervous system has rarely been reported.

Our previously published study showed that krill oil alleviated oxidative stress and apoptosis induced by METH *in vitro* (Xiong et al., 2018). Therefore, the aim of the present study was to evaluate the protective potential of krill oil in mice subjected to chronic METH exposure *in vivo*. Network pharmacology is an excellent approach for the study of multicomponent compounds through multi-target and multi-pathway therapeutic mechanisms. Therefore, after the behavioral test, network pharmacological tools and resources were used to screen the potential targets and pathways of major active components of krill oil and to reveal their mechanism of action in the treatment of METH-induced memory impairment. In addition, experiments were also conducted to validate the potential underlying mechanism of krill oil on METH-induced memory impairment, as predicted by the network pharmacology approach.

## Materials and Methods

### Reagents

METH (98%) was offered by the Hubei Public Security Bureau. Krill oil was provided by the Aker BioMarine Antarctic Company (Norway). Antibodies against protein kinase A (PKA), phosphorylated protein kinase A (p-PKA), cAMP-response element-binding protein (CREB), phosphorylated CREB (p-CREB), extracellular regulated protein kinase 1/2 (ERK1/2), and phosphorylated ERK1/2 (p-ERK1/2) were bought from Cell Signaling Technology (Danvers, United States). Antibodies against the dopamine D1 receptor (DRD1), dopamine D2 receptor (DRD2), dopamine transporter (DAT), and cleaved caspase-3 were obtained from Absin Bioscience Co., Ltd. (Shanghai, China). Antibodies against GAPDH, horseradish peroxides (HRP)-conjugated goat anti-rabbit antibody and HRP-conjugated goat anti-mouse antibody, protein extraction buffer, protease inhibitors, and phosphatase inhibitors were obtained from Wuhan Boster Biological Technology Co., Ltd. (Wuhan, China). Antibodies against the brain-derived neurotrophic factor (BDNF) were purchased from Santa Cruz Biotechnology (Santa Cruz, United States). All other reagents used were of analytical grade.

### Animal Treatment

**FIGURE 1:**
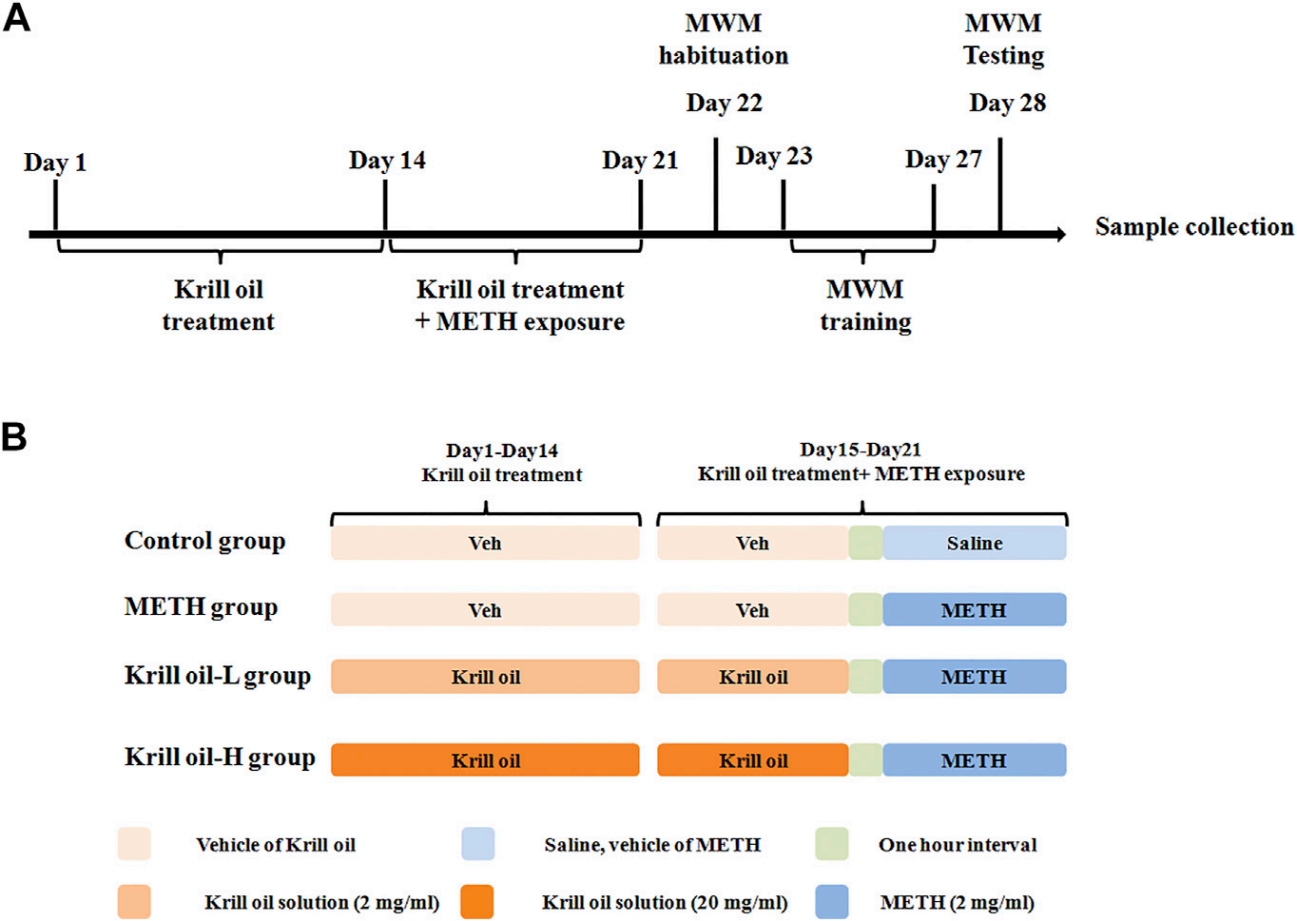
Experimental procedure of krill oil–ameliorated memory deficits induced by METH. **(A)** For the first 3 weeks, mice were administrated with different doses of krill oil with or without METH. After that, the behavioral tests and Western blotting experiments were performed. **(B)** Drug treatment of the animal experiment. For the first 2 weeks, mice were intragastrically administrated with different doses of krill oil or vehicle. At the third week, 1 h after the intragastric administration, mice were intraperitoneally injected with METH or saline.

C57BL/6 mice (male, 8 weeks) were provided by Beijing Vital River Laboratory Animal Technology Co., Ltd. Mice were housed five per cage in a 12-h light–dark cycle and a temperature-controlled environment. All animal experiment procedures were approved by the Ethics Committee of Jianghan University. Specifically, mice were divided randomly into the control group, METH group, krill oil-L group and krill oil-H group, and the experimental procedure is detailed in Figure 1A. Before administration, krill oil was dissolved in ethanol and then diluted with saline, and METH was dissolved in saline at a concentration of 10 mg/ml. For the first 2 weeks, mice in the krill oil-L group and krill oil-H group were intragastrically administrated with 10 mg/kg and 100 mg/kg krill oil every day, respectively, and mice in the control group and METH group were intragastrically administrated with vehicle. At the third week, 1 hour after intragastric administration, mice in the METH, krill oil-L group, and krill oil-H group were intraperitoneally injected with 10 mg/kg METH, and mice in the control group were intraperitoneally injected with saline. Behavioral experiments, sample collection, and the Western blot test were performed as follows (Figure 1).

### Morris Water Maze Task

Memory training on the hidden platform of the Morris water maze was used to measure the associative, spatial memory of mice as a previous report. Briefly, water (23 ± 2°C) was added into a circular pool (21 cm in deep and 120 cm in diameter). A circular hidden platform was placed in the center of the target quadrant and submerged 1.5 cm below the water surface. XR-XM101 software was used to automatically measure the animal escape latency, swimming speed, and the amount of time spent in the target quadrant (Shanghai Xinruan Information Technology Co., Ltd.). Mice underwent four trials each day during the training phase, and the starting position of each trial was different. Mice have a maximum of 60 s in each trial to find the platform, and mice were allowed to stay on the platform for 15 s after boarding the platform. If the mice cannot find the platform within 60 s, it was guided to the platform to rest for 15 s. The time interval between trials was 30 min. Mice were dried with a towel, placed in a cage with a heating lamp, and then returned to their home cage after the last trial. The training phase lasted for 5 days. To test the spatial memory ability of mice, a 60-s free swimming test without a platform was performed on the sixth day.

### Network Pharmacology Analysis

The potential protein targets of krill oil, related genes of METH, and related genes of memory deficits were collected from the GeneCards database (https://www.genecards.org/). Then, the protein targets of krill oil were mapped with related genes of METH and related genes of memory impairment on the Bioinformatics and Evolutionary Genomics website (http://bioinformatics.psb.ugent.be/webtools/Venn/). To further characterize the molecular mechanism of krill oil on METH-induced memory deficits, the compound–target networks were generated using Cytoscape 3.8.0. In these graphical networks, the compounds and proteins were expressed as nodes, whereas the compound–target interactions were expressed as edges.

The gene ontology (GO) analyses and KEGG pathway analyses were conducted using the functional annotation tool of DAVID Bioinformatics Resources 6.7 (http://david.abcc.ncifcrf.gov/). Terms with thresholds of counts ≥ 10 and *p* values ≤ 0.05 were chosen in functional annotation clustering. Related target proteins of krill oil on METH-induced memory deficits were analyzed by online STRING 11.0 (https://string-db.org/) to construct a protein–protein interaction (PPI) network. The network was visualized with Cytoscape (v3.1.2) and CytoHubba, a plug-in in Cytoscape, to filter the modules from the PPI network and to obtain the most important hub genes based on the degree score.

### Western Blotting Analysis

Hippocampi were isolated, lysed, and centrifuged for 15 min (4°C) at 12,000 g . After the detection of concentration, the supernatants were mixed with a loading buffer and denatured for 5 min. Protein samples were separated using a gel electrophoresis system and transferred to the polyethylene difluoride membranes. After blocking for 1 h in 5% non-fat milk, the membranes were incubated with the primary antibody and then with the HRP secondary antibody. Enhanced chemiluminescence (Thermo Fisher, United States) was used to observe the bands using a chemiluminescence detector (Gene Corporation, Hong Kong). The intensity of each band was determined quantitatively by ImageJ and calibrated by the corresponding internal reference protein, and the results were shown as normalized for the control group.

### Statistical Analysis

Data were expressed as the mean ± standard error (SEM). All results were analyzed using SPSS 23.0 software. Results of the swimming speed and escape latency during the training phase of MWM were analyzed by one-way ANOVA with repeated measures. Other data used one-way ANOVA and Tukey’s HSD *post hoc* test. A *p* value less than 0.05 was considered statistically significant.

## Results

### Krill Oil Ameliorated the Impairment of Spatial Learning and Memory Induced by METH in MWM Task

**FIGURE 2:**
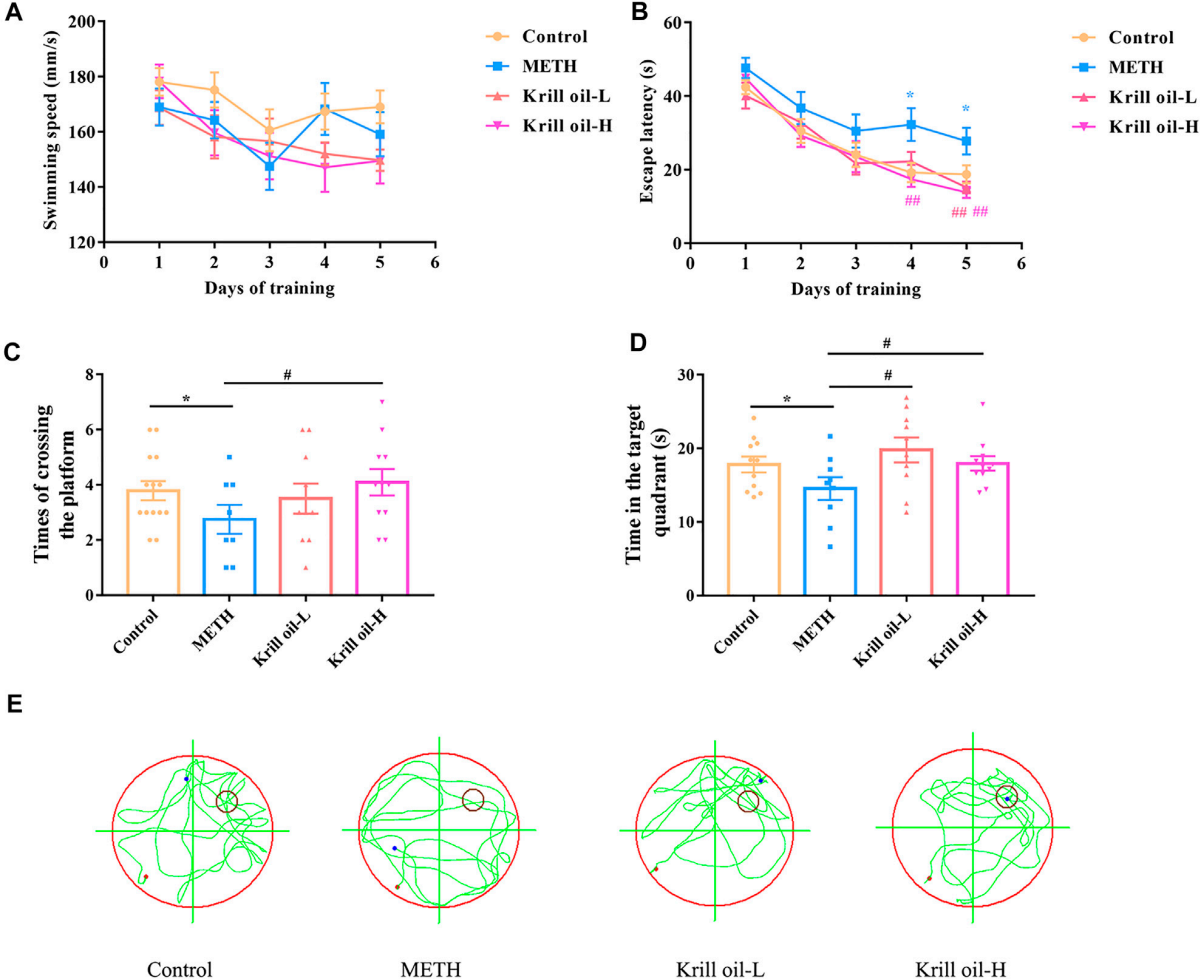
Krill oil–ameliorated impairment of spatial learning and memory induced by METH in the MWM task. **(A)** Swimming speed of each group during the training session. The Morris water maze test was conducted to evaluate spatial learning and memory function. **(B)** Acquisition of spatial memory in mice. The escape latency during the training phase was shown. **(C)** Retention of spatial memory in mice. Times of crossing the platform of mice were shown. **(D)** Retention of spatial memory in mice. The time in the target quadrant of mice was shown. **(E)** Representative swimming trajectories of Morris water maze testing. *n* = 10 for each group, and data were presented by means ± SEM, *p < 0.05, **p < 0.01 compared to the control group, and #*p* < 0.05, ##*p* < 0.01 compared to the METH group.

The Morris water maze test was used to determine whether krill oil alleviated METH-induced spatial learning and memory impairment. As shown in Figure 2, the locomotor activity of mice was not influenced by METH or krill oil treatment since the swimming speed did not differ among the groups (Figure 2A). Mice in the METH group had a higher escape latency than those in the control group on day 4 and day 5 of the training phase (Figure 2B), suggesting that repeated METH exposure triggered a decline in the spatial learning ability of mice (*p* < 0.05). Moreover, compared with the METH group, 10 mg/kg krill oil treatment greatly reduced the increase of learning latency in mice induced by METH on day 5 (*p* < 0.01), and mice pretreated with 100 mg/kg of krill oil performed significantly better than those that received METH alone on day 4 and day 5 (*p* < 0.01), suggesting that learning deficits were improved following krill oil treatment. In the testing section, results revealed significant differences among group effects (Figures 2C,D, *p* < 0.05). Mice in the METH group had worse performance in the parameter of times of crossing the platform and time in the quadrant of the platform than those of the control group (*p* < 0.05). Treatment with krill oil (10 or 100 mg/kg), however, remarkably increased the times of crossing the platform and the time in the quadrant of the platform (*p* < 0.05). The swimming trajectory further confirmed that the krill oil–treated mice stayed in the target quadrant longer than the METH group (Figure 2E). Taken together, these findings demonstrated that krill oil improved METH-induced cognitive deficits of spatial learning and memory.

### Target Identification of Krill Oil on METH-Induced Memory Deficits

**FIGURE 3:**
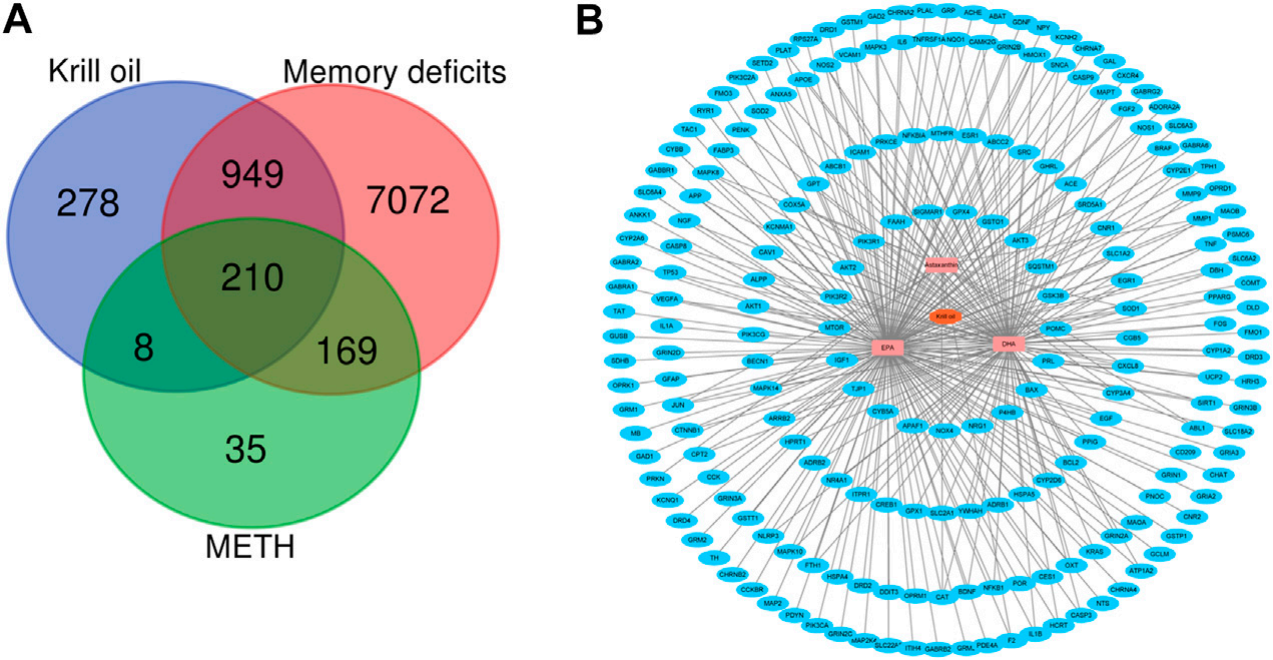
Compound–target network for krill oil on METH-induced memory deficits. **(A)** Venn plot for the possible targets of main compounds of krill oil and related genes of METH and memory deficits. **(B)** Network of active compounds and the possible targets for krill oil on METH-induced memory deficits. The orange polygon represented krill oil. The pink squares represented active compounds of krill oil and the blue circle represented potential protein targets. The edges represent the interactions between them.

Krill oil from *Euphausia superba* (Antarctic krill), an Antarctic marine species, is rich in EPA, DHA, and astaxanthin. Among the three main bioactive components of krill oil, 1,846 protein targets were retrieved from the GeneCards database. The detailed information is shown in Supplementary Table S1. After eliminating the overlaps, 1,445 protein targets were obtained for further analyses. 8400 memory deficit–related genes and 422 METH-related genes were collected from the GeneCards database. The detailed information is shown in Supplementary Tables S2, and S3. Then, these protein targets of krill oil were mapped with related genes of METH and memory impairment on the Bioinformatics and Evolutionary Genomics website. As a result, 210 targets of krill oil were associated with METH-induced memory impairment, and the detailed information of the 210 targets is shown in Supplementary Table S4 and Figure 3A. Among the 210 target genes, 116 were target genes for DHA, 174 were target genes for EPA, and 16 were target genes for astaxanthin. The detailed information is shown in Supplementary Table S5 and Supplementary Figure S1.

As krill oil may exhibit multiple pharmacological activities *via* multiple targets, it was constructive to investigate the underlying mechanisms of krill oil on complex diseases by network analysis. In the current study, the compound–target network of krill oil on METH-induced memory deficits was constructed with Cytoscape 3.8.1 software (Figure 3B). Among these potential protein targets, there were 12 high-degree targets associated with multiple compounds (Supplementary Figure S1), namely, NFKBIA, COX5A, MAPK14, HMOX1, NOS2, MAPK8, JUN, CAT, MMP1, CYP3A4, CXCL8, and SOD1. These high-degree protein targets in the network may account for the essential protective effects of krill oil on METH-induced memory impairment.

### GO and Pathway Enrichment Analyses

**FIGURE 4:**
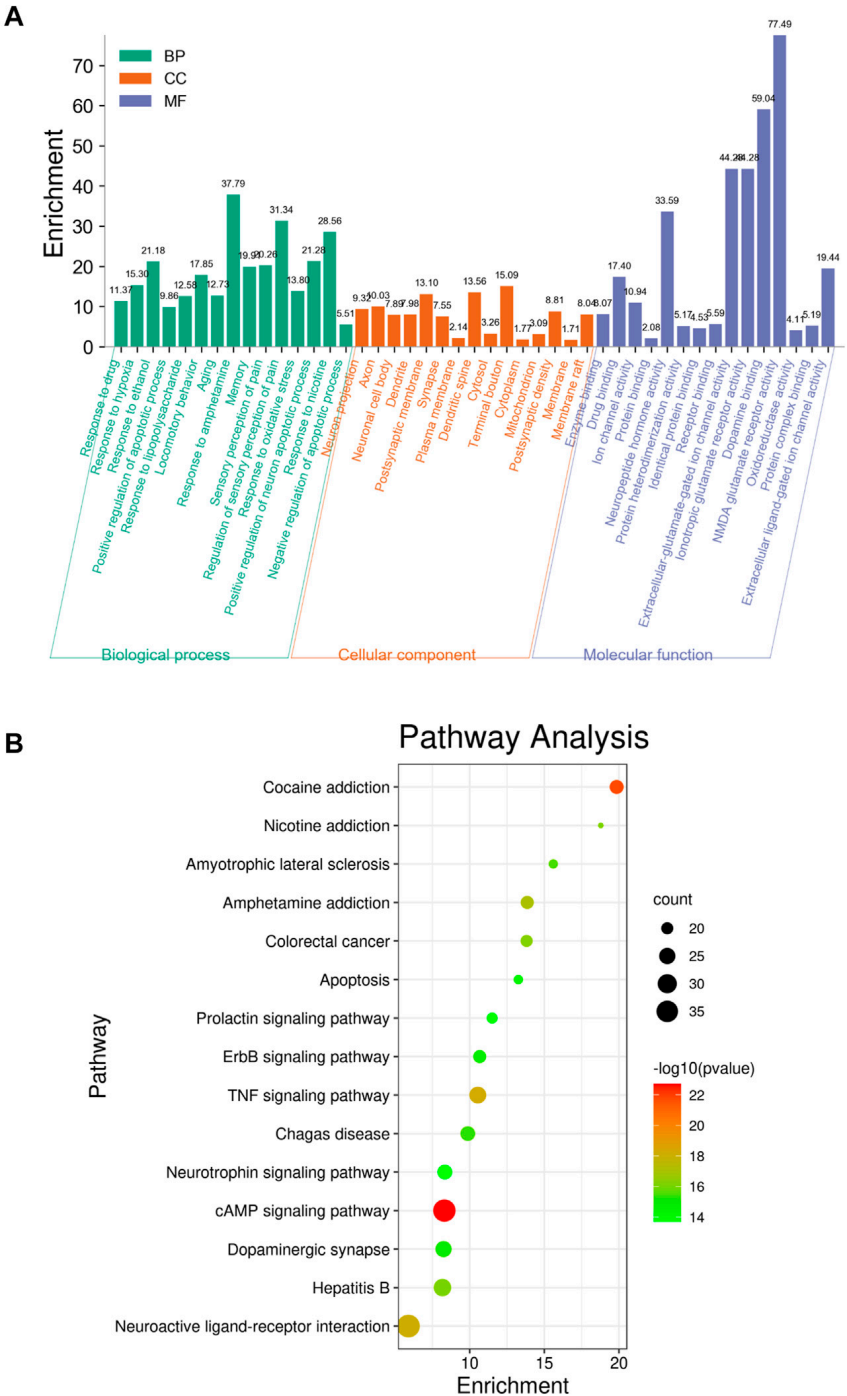
Top 15 significantly enriched terms gene ontology **(A)** and pathway enrichment **(B)** analyses of potential target genes of krill oil on METH-induced memory deficits.

To identify the biological characteristics of putative targets of krill oil on METH-induced memory impairment in detail, the GO and KEGG pathway enrichment analyses of involved targets were conducted *via* the functional annotation tool of DAVID Bioinformatics Resources 6.8. There were 78 biological processes (BP), 46 cellular components (CC), and 23 molecular function (MF) terms in total, which met the requirements of counts ≥ 10 and *p* values ≤ 0.05. The detailed GO information is shown in Supplementary Table S6. The top 15 significantly enriched terms in BP, CC, and MF categories are shown in Figure 4A, which indicated that krill oil may improve METH-induced memory impairment *via* regulation of the neuron apoptotic process, response to oxidative stress, response to hypoxia, dopamine binding, NMDA glutamate receptor activity, and oxidoreductase activity. To explore the underlying involved pathways of krill oil on METH-induced memory impairment, a KEGG pathway analysis of involved targets was conducted. The detailed pathway information of krill oil on METH-induced memory impairment is shown in Supplementary Table S7. There were 94 pathways that met the requirements of counts ≥ 10 and *p* values ≤ 0.05. The top 15 significantly enriched pathways are shown in Figure 4B. The pathways in neuroactive ligand–receptor interaction exhibited the largest number of involved targets (38 counts).

### Screen of Hub Genes

**FIGURE 5:**
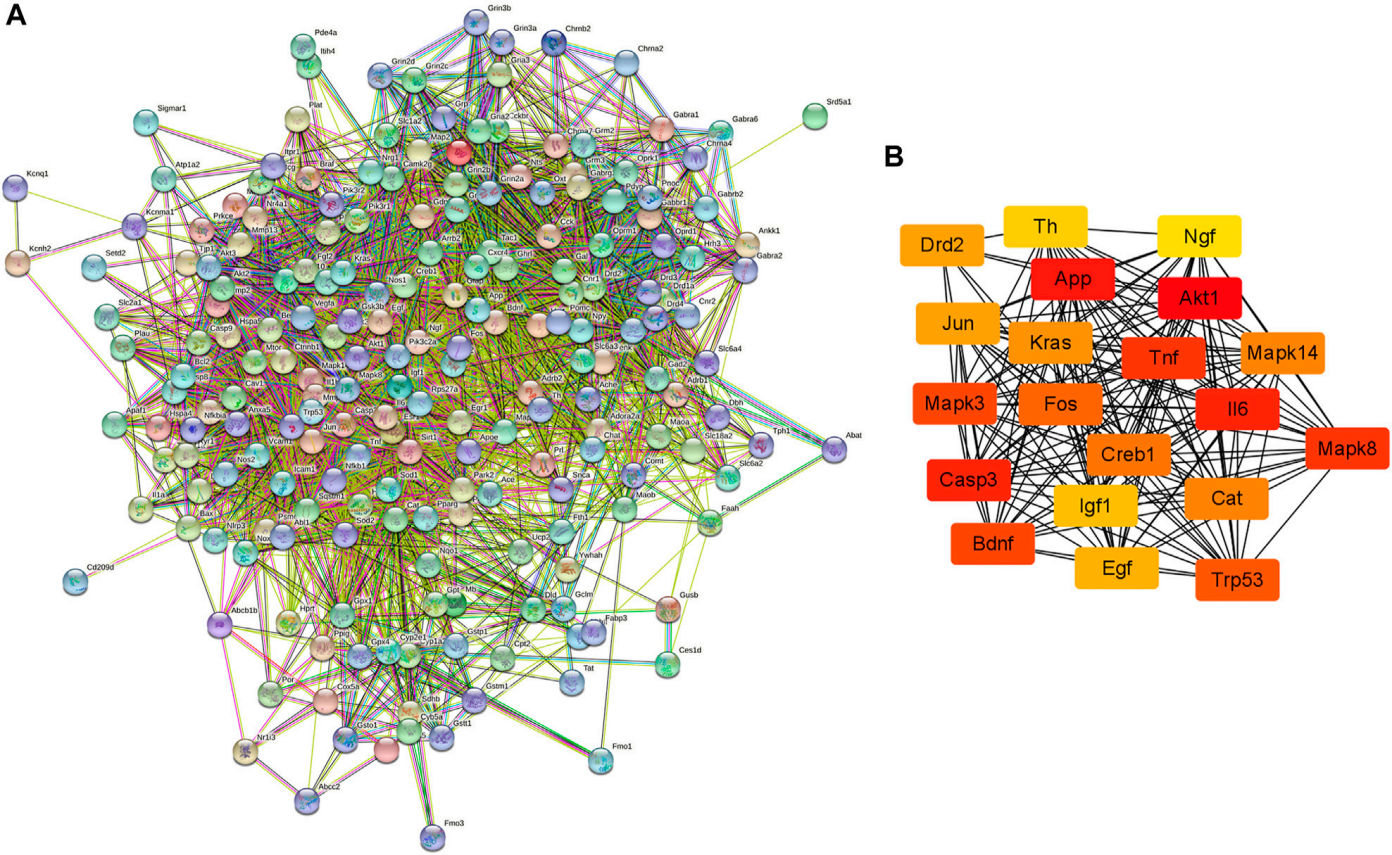
PPI network of the potential target genes and hub genes screen. **(A)** The PPI network of the potential target genes was constructed using STRING. **(B)** The most significant hub genes were obtained from the PPI network using Cytoscape and CytoHubba.

The PPI network of possible target genes involved in the protective effect of krill oil on METH-induced memory impairment was constructed using STRING (Figure 5A).

**FIGURE 6:**
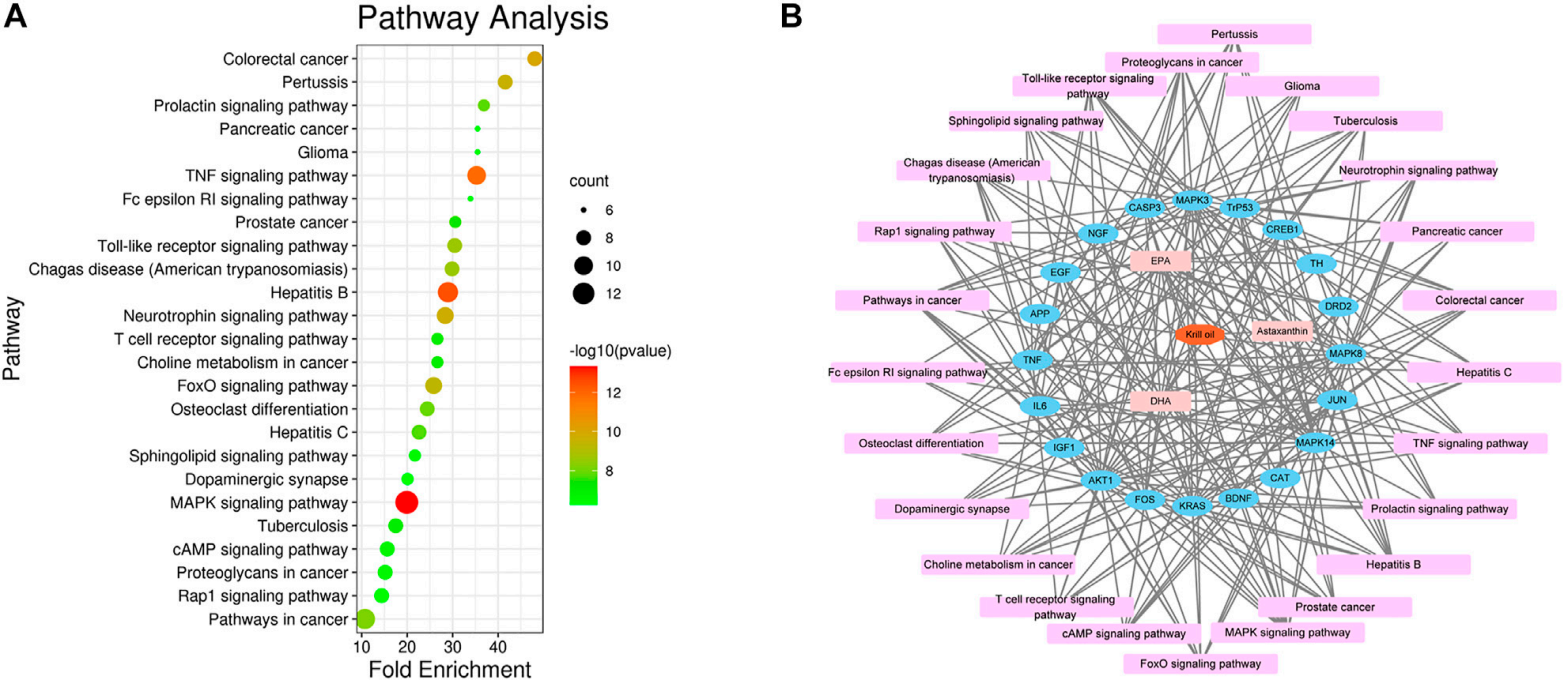
Pathway enrichment and network analyses of hub genes. **(A)** The top 25 significantly enriched pathways of hub genes. **(B)** The compound–target–pathway network for krill oil on METH-induced memory deficits. The orange polygon represented krill oil. The pink squares represented active compounds of krill oil and the blue circle represented potential hub genes. The mauve squares represented pathways. The edges represent the interactions between them.

There were 205 nodes and 3,388 edges, and the average node degree was 33.1. The average local clustering coefficient was 0.565, and the *p* value of PPI enrichment was less than 1.0e-16. To obtain the hub genes in the PPI network, these node pairs were entered into Cytoscape software. The scores of nodes were calculated by CytoHubba, and the top 20 hub genes are shown in Figure 5B, and the hub gene symbols, full names, and functions are shown in Table 1. The pathway enrichment analyses of hub genes were conducted *via* DAVID Bioinformatics Resources 6.8. There were 54 pathways that met the requirements of count ≥ 5 and *p* values ≤ 0.05. The detailed pathway information of hub genes is shown in Supplementary Table S8. The top 25 significantly enriched pathways are shown in Figure 6A and Table 2. The compound–hub gene–pathway network of krill oil on METH-induced memory deficits was constructed with Cytoscape 3.8.1 software (Figure 6B).

**TABLE 1:** Detail information of hub genes involved in the neuroprotective effect of krill oil on METH-induced memory impairment.

Hub gene symbol	Full names	Functions	Degree
AKT1	RAC-alpha serine/threonine-protein kinase	Regulates many processes including metabolism, proliferation, cell survival, growth, and angiogenesis	117
APP	Amyloid-beta precursor protein	A cell surface receptor and performs physiological functions on the surface of neurons relevant to neurite growth, neuronal adhesion, and axonogenesis	95
IL6	Interleukin-6	A cytokine with a wide variety of biological functions in immunity, tissue regeneration, and metabolism	92
CASP3	Caspase-3	Involved in the activation cascade of caspases responsible for apoptosis execution	92
MAPK8	Mitogen-activated protein kinase 8	Serine/threonine-protein kinase involved in various processes such as cell proliferation, differentiation, migration, transformation, and programmed cell death	91
TNF	Tumor necrosis factor	It is a cytokine and mainly secreted by macrophages. It can induce cell death of certain tumor cell lines and bind to TNFRSF1A/TNFR1 and TNFRSF1B/TNFBR	91
BDNF	Brain-derived neurotrophic factor	Promotes the survival and differentiation of selected neuronal populations of the peripheral and central nervous systems during development	90
MAPK3	Mitogen-activated protein kinase 3	MAPK1/ERK2 and MAPK3/ERK1 are the 2 MAPKs which play an important role in the MAPK/ERK cascade and act as essential components of the MAP kinase signal transduction pathway	90
TRP53	Cellular tumor antigen p53	Acts as a tumor suppressor in many tumor types, induces growth arrest, or apoptosis depending on the physiological circumstances and the cell type	87
FOS	Proto-oncogene c-Fos	A nuclear phosphoprotein which forms a tight but non-covalently linked complex with the JUN/AP-1 transcription factor	83
CREB1	Cyclic AMP-responsive element-binding protein 1	Phosphorylation-dependent transcription factor that stimulates transcription upon binding to the DNA cAMP response element (CRE), a sequence present in many viral and cellular promoters	80
CAT	Catalase	Occurs in almost all aerobically respiring organisms and serves to protect cells from the toxic effects of hydrogen peroxide	79
MAPK14	Mitogen-activated protein kinase 14	MAPK14 is one of the four p38 MAPKs which play an important role in the cascades of cellular responses evoked by extracellular stimuli such as pro-inflammatory cytokines or physical stress leading to direct activation of transcription factors	79
KRAS	GTPase KRas	Ras proteins bind GDP/GTP and possess intrinsic GTPase activity and play an important role in the regulation of cell proliferation	74
DRD2	D(2) dopamine receptor	Dopamine receptor whose activity is mediated by G proteins which inhibit adenylyl cyclase	72
JUN	Transcription factor AP-1	A transcription factor that recognizes and binds to the enhancer heptamer motif 5′-TGA[CG]TCA-3'	72
EGF	Pro-epidermal growth factor	EGF stimulates the growth of various epidermal and epithelial tissues *in vivo* and *in vitro* and of some fibroblasts in the cell culture	71
IGF1	Insulin-like growth factor I	The insulin-like growth factor is structurally and functionally related to insulin but have a much higher growth-promoting activity	70
TH	Tyrosine 3-monooxygenase	Plays an important role in the physiology of adrenergic neurons	69
NGF	Beta-nerve growth factor	Nerve growth factor is important for the development and maintenance of the sympathetic and sensory nervous systems	67

**TABLE 2:** Top 25 significantly enriched pathways of hub genes.

Number	Pathway	Genes
1	MAPK signaling pathway	JUN, BDNF, EGF, FOS, NGF, MAPK14, TNF, MAPK8, TRP53, CASP3, AKT1, KRAS, and MAPK3
2	Hepatitis B	IL6, MAPK8, JUN, CREB1, TRP53, CASP3, AKT1, KRAS, FOS, TNF, and MAPK3
3	TNF signaling pathway	IL6, MAPK8, JUN, CREB1, CASP3, AKT1, FOS, MAPK14, TNF, and MAPK3
4	Colorectal cancer	MAPK8, JUN, TRP53, CASP3, AKT1, KRAS, FOS, and MAPK3
5	Neurotrophin signaling pathway	MAPK8, JUN, BDNF, TRP53, AKT1, KRAS, MAPK14, NGF, and MAPK3
6	Pertussis	IL6, MAPK8, JUN, CASP3, FOS, MAPK14, TNF, and MAPK3
7	FoxO signaling pathway	IL6, MAPK8, EGF, CAT, AKT1, KRAS, IGF1, MAPK14, and MAPK3
8	Toll-like receptor signaling pathway	IL6, MAPK8, JUN, AKT1, FOS, MAPK14, TNF, and MAPK3
9	Chagas disease (American trypanosomiasis)	IL6, MAPK8, JUN, AKT1, FOS, MAPK14, TNF, and MAPK3
10	Pathways in cancer	IL6, MAPK8, JUN, EGF, TRP53, CASP3, AKT1, KRAS, IGF1, FOS, and MAPK3
11	Osteoclast differentiation	MAPK8, JUN, CREB1, AKT1, FOS, MAPK14, TNF, and MAPK3
12	Prolactin signaling pathway	MAPK8, TH, AKT1, KRAS, FOS, MAPK14, and MAPK3
13	Hepatitis C	MAPK8, EGF, TRP53, AKT1, KRAS, MAPK14, TNF, and MAPK3
14	Prostate cancer	CREB1, EGF, TRP53, AKT1, KRAS, IGF1, and MAPK3
15	Choline metabolism in cancer	MAPK8, JUN, EGF, AKT1, KRAS, FOS, and MAPK3
16	T-cell receptor signaling pathway	JUN, AKT1, KRAS, FOS, MAPK14, TNF, and MAPK3
17	Tuberculosis	IL6, MAPK8, CREB1, CASP3, AKT1, MAPK14, TNF, and MAPK3
18	cAMP signaling pathway	MAPK8, JUN, CREB1, BDNF, AKT1, FOS, DRD2, and MAPK3
19	Proteoglycans in cancer	TRP53, CASP3, AKT1, KRAS, IGF1, MAPK14, TNF, and MAPK3
20	Sphingolipid signaling pathway	MAPK8, TRP53, AKT1, KRAS, MAPK14, TNF, and MAPK3
21	Pancreatic cancer	MAPK8, EGF, TRP53, AKT1, KRAS, and MAPK3
22	Glioma	EGF, TRP53, AKT1, KRAS, IGF1, and MAPK3
23	Rap1 signaling pathway	EGF, AKT1, KRAS, IGF1, MAPK14, NGF, DRD2, and MAPK3
24	Fc epsilon RI signaling pathway	MAPK8, AKT1, KRAS, MAPK14, TNF, and MAPK3
25	Dopaminergic synapse	MAPK8, CREB1, TH, AKT1, FOS, MAPK14, and DRD2

The pathways in the MAPK signaling pathway exhibited the largest number of involved targets (13 counts). Among the 20 hub genes, 17 were target genes for DHA, 17 were target genes for EPA, and 4 were target genes for astaxanthin. The detailed information is shown in Supplementary Table S9 and Supplementary Figure S2.

### Expression Levels of Hub Genes in the Hippocampus of Mice

**FIGURE 7:**
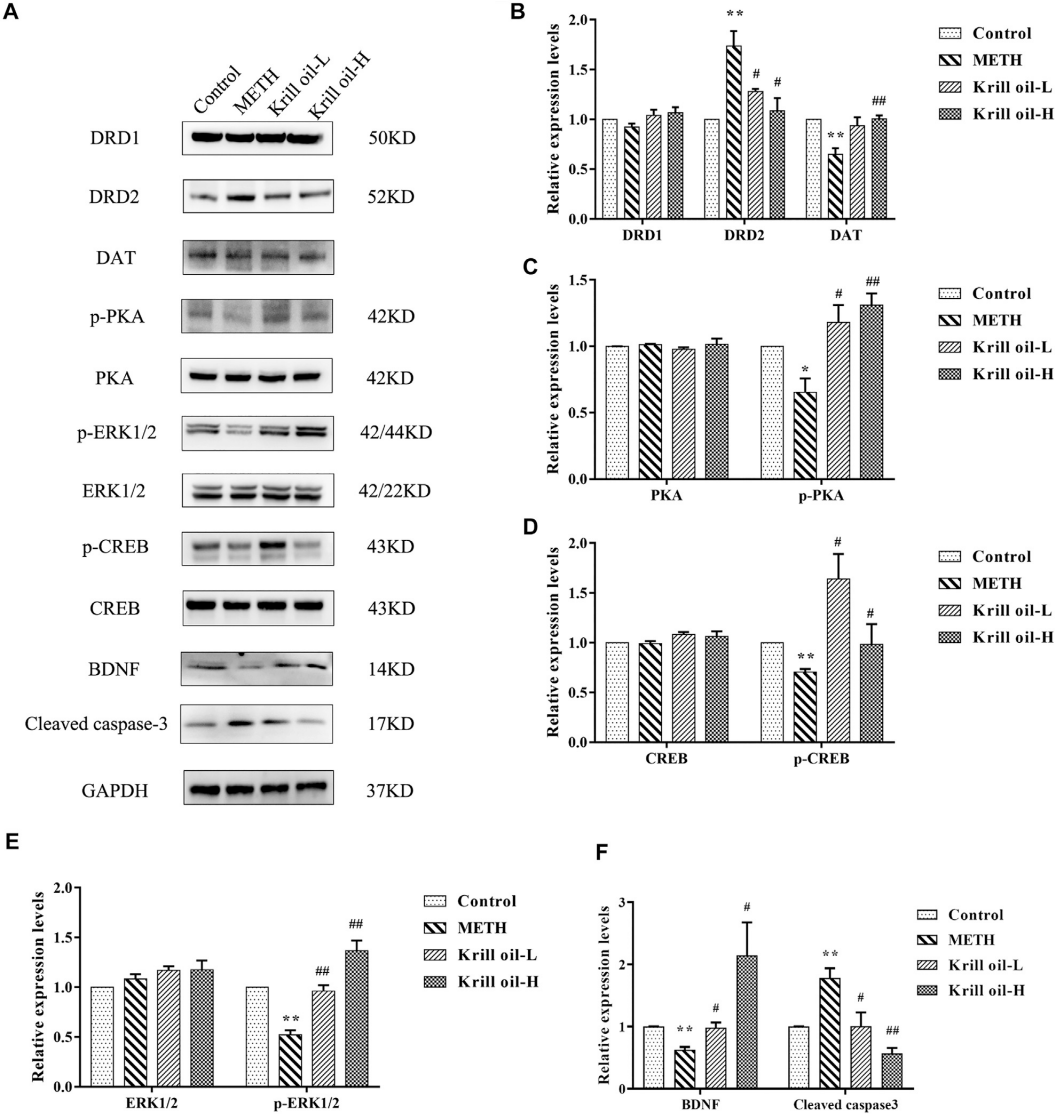
Relative expressions of related proteins in the hippocampus of mice treated with METH and/or krill oil. **(A)** Representative images of DRD1, DRD2, DAT, p-PKA, PKA, p-ERK1/2, ERK1/2, p-CREB, CREB, BDNF, and cleaved caspase-3. **(B)** Statistical results of DRD1, DRD2, and the DAT protein. **(C)** Statistical results of the p-PKA and PKA protein. **(D)** Statistical results of the p-CREB and CREB protein. **(E)** Statistical results of the p-ERK1/2 and ERK1/2 protein. **(F)** Statistical results of the mature BDNF protein and cleaved caspase-3. Data were presented as means ± SEM. *p < 0.05, **p < 0.01 compared to the control group, #*p* < 0.05, ##*p* < 0.01 compared to the METH group.

Network pharmacology analysis predicted that the molecular targets highly associated with the common signaling pathways including the MAPK signaling pathway, dopaminergic synapse, and cAMP signaling pathway may be related to the neuroprotective effect of krill oil on METH-induced memory impairment in regulating neuron functions and apoptosis. We further validated the expressions of the potential hub genes identified *via* network pharmacology. As shown in Figures 7A and B, compared with the control group, the expression of DRD2 in the hippocampus of mice in the METH group was greatly increased and the expression of DAT was decreased, and 100 mg/kg krill oil treatment significantly decreased the expression of DRD2 and increased the expression of DAT, while 10 mg/kg krill oil treatment significantly decreased the expression of DRD2. There was no significant difference of the expression of DRD1 among different groups. Likewise, METH treatment led to apparent repression of p-PKA, p-ERK1/2, p-CREB, and BDNF (Figures 7A,C–F), and 10 and 100 mg/kg krill oil treatment significantly increased the expression of p-PKA, p-ERK1/2, p-CREB, and BDNF. There were no significant differences of the expression of PKA, ERK1/2, and CREB among different groups. Moreover, METH treatment led to apparent enrichment of cleaved caspase-3 (Figures 7A,F), and 10 and 100 mg/kg krill oil treatment significantly reduced the expression of cleaved caspase-3. These results validated that krill oil may regulate the neuron functions and apoptosis mainly through the MAPK signaling pathway, cAMP signaling pathway, and dopaminergic synapse pathway.

## Discussion

Krill oil, extracted from small Antarctic krill, has been reported to have health benefits, including improved memory impairment, systemic inflammation, glucose metabolism, and hepatic steatosis (Kwantes and Grundmann 2015; Sistilli et al., 2021; Zhou et al., 2021). This study aimed to assess the ability of krill oil supplementation to affect memory dysfunction induced by METH in mice and figure out the possible mechanism. In the present study, we found that krill oil enhanced the neurocognitive functions of METH-treated mice, and targets like DRD2, MAPK, CREB, CASP3, and BDNF, and some signaling pathways such as the MAPK signaling pathway, PI3K-Akt signaling pathway, AMPK signaling pathway, neurotrophin signaling pathway, and cAMP signaling pathway were filtered out as the underlying mechanisms of krill on improving memory deficits induced by METH. To our best knowledge, it is the first time that integrating system pharmacology and bioinformatics analysis have been used to predict mechanisms of krill oil in the treatment of central nervous system injury.

Multiple METH exposures can result in neurodegenerative changes, including long-term cognitive dysfunction, memory deficits, and depression in humans and animal models (Ru et al., 2019; Zeng et al., 2021). The data from the literature revealed that krill oil supplementation in the diet could suppress neuroinflammation, oxidative stress, and improve the lipopolysaccharide-induced cognitive impairment (Polotow et al., 2015; Choi et al., 2017). In this study, we investigated the effect of krill oil on METH-induced memory impairment and discovered the possible molecular mechanism. The MWM task was used to investigate the ability of mice to learn locations and perform spatial memory recall through escape latency and measuring times to cross the platform in the water maze. The results of the MWM task showed that METH-treated mice learned more slowly than mice in the control group during the training period, and 10 mg/kg krill oil pretreatment significantly reduced escape latency on day 5 of the training phase, while 100 mg/kg krill oil pretreatment significantly reduced escape latency on days 4 and 5. For testing the maintenance of memory, the times of crossing the platform and the time spent in the target quadrant in the METH group were significantly decreased, and 10 mg/kg krill oil–treated mice spent much more time in the target quadrant zone than METH-treated mice, while mice in the 100 mg/kg krill oil group had more probability of crossing the platform and target quadrant time. Thus, pretreatment with krill oil could improve neurocognitive functions and alleviate the impairment of learning and memory caused by METH, and the protective effect of krill oil may be improved by increasing the dose within the dose range of 10–100 mg/kg.

Krill oil consists of multiple components, so it is difficult to determine the molecular mechanisms with traditional technology. Network pharmacology methods provide an effective tool for investigating multicomponents and exploring the mechanisms (Zhou et al., 2021). To determine the molecular mechanisms of krill oil on METH-induced memory impairment, network pharmacology approaches were used in this study. We selected DHA, EPA, and astaxanthin, which were present in high contents in krill oil, as the main active compounds, and the GeneCards database was used to identify the potential targets of krill oil, memory deficits, or METH. After the evaluation of the primary network nodes and potential targets, 210 targets were identified in the drug–compound–target network, which indicated that krill oil had multi-targets that were involved in the regulation of multiple signaling pathways. With the use of the CytoHubba and MCODE analysis, 20 genes were identified as hub genes. From the integrated hub target prediction and pathway analysis, krill oil may exert its neuroprotective effects on METH-induced memory deficits *via* the regulation of neuron functions and apoptosis, which was characterized as the important mechanism of memory impairment (Muhammad et al., 2019; Skelly et al., 2019).

As predicted by network pharmacology methods, CASP3, BDNF, MAPK3, CREB1, and DRD2 were the hub genes in krill oil to alleviate METH-induced memory impairment. KEGG signaling pathway enrichment analyses showed that krill oil may exert therapeutic effects on memory deficits primarily by regulating neuron and cell apoptosis *via* the MAPK signaling pathway, dopaminergic synapse, and cAMP signaling pathway. To further validate the postulation, we investigated the expression levels of main hub genes. Dopamine neurotransmission is critical for the physiological activity of the brain, including spatial learning and psychiatric disorders (Kempadoo et al., 2016). Because of its structural similarity to dopamine, METH could act on the dopamine transporter (DAT) in the presynaptic membrane, which inhibits dopamine reuptake and increases the dopamine level in the synaptic cleft, thereby causing overactivation of dopamine receptors in the postsynaptic membrane and excitatory oxidative damage to postsynaptic neurons (Ares-Santos et al., 2013; Yu et al., 2015). There are five types of dopamine receptors grouped into two major subclasses: DRD1-like, including DRD1 and D1RD5, and DRD2-like, including DRD2, DRD3, and DRD4, which often interact to regulate neurotransmission (Sun et al., 2017). Repeated METH exposure could increase the protein expression of DRD2 in the hippocampus area and reduce the protein expression of DAT (Zhou et al., 2019), and pretreatment with DRD2 antagonist sulpiride attenuated the effects of METH on egocentric and spatial learning and memory (Gutierrez et al., 2019). DAT-KO rats demonstrated deficits in sensorimotor gating and working memory tests (Leo et al., 2018). In line with the literature, our results showed that the expressions of DRD2 receptors in the hippocampus were greatly increased, while the levels of DAT were reduced after repeated METH exposure. Meanwhile, 100 mg/kg krill oil pretreatment significantly decreased the expressions of DRD2 and increased the expressions of DAT. The expression levels of DRD1, which was not a hub gene, did not change among groups. These results confirmed the results predicted by network pharmacology and validated the role of the dopaminergic synapse pathway in the improvement of METH-induced memory impairment by krill oil.

MAP kinases (MAPK), also known as extracellular signal-regulated kinases (ERK), are involved in a variety of biochemical processes such as cell proliferation and differentiation. MAPK3/ERK1 is one of the important members of the MAP kinase family. The cAMP-response element binding protein (CREB), which has been reported to be involved in the learning and memory deficits, is the target of ERK1/2, and protein kinase A (PKA) also can mediate the increase in ERK1/2 phosphorylation (Lyu et al., 2020; Mu et al., 2020). Therefore, after METH exposure, the expressions of p-PKA, p-ERK1/2, and p-CREB were significantly reduced. When mice were pretreated with 10 and 100 mg/kg krill oil, the increased expression of p-PKA, p-ERK1/2, and p-CREB may partially contribute to its improved effects of memory impairment. The brain-derived neurotrophic factor (BDNF) is a key factor of synaptic transmission and plays an important role in supporting neuronal survival, regulating synaptogenesis and contributing to the formation of memory (Leal et al., 2014). As a transcription factor, CREB regulates the transcription of BDNF, especially the activation of BDNF promoters I and IV (Esvald et al., 2020). From the experimental validation, our results showed that METH may decrease BDNF mostly by lowering the phosphorylated CREB protein. Krill oil may raise the levels of BDNF by activating the ERK1/2/CREB signaling pathway. Taken together, our findings suggested that krill oil may improve METH-induced memory deficits mainly *via* the regulation of neuron functions and apoptosis through the MAPK, dopaminergic synapse, and cAMP signaling pathway.

Hub gene caspase-3 is a key cysteine protease protein and acts as one of the executioner caspases (caspase-3, -6, and -7) that carry out the demolition phase of apoptosis (Boice and Bouchier-Hayes 2020). Cleaved caspase-3 can cleave structural and functional proteins in cells and induce cell apoptosis (Jiang et al., 2020). METH induced increase in the expression of cleaved caspase-3 in the hippocampus, which was involved in METH-induced neurotoxicity as well as spatial learning and memory impairments (Wen et al., 2019). Our previous data showed that krill oil could inhibit the METH-induced increase of cleaved caspase-3 *in vitro* (Xiong et al., 2018)*.* Caspase-3 was one of the predicted hub gene; herein, we investigated the expression of cleaved caspase-3. Results showed that the expressions of cleaved caspase-3 were significantly increased after METH exposure, and pretreatment with both 10 and 100 mg/kg krill oil reduced the increasing expression of cleaved caspase-3, which may partially contribute to its improved effects of memory impairment.

## Conclusion

In summary, the neuroprotective effect of krill oil on METH-induced memory impairment and the underlying pharmacological mechanism were investigated with the combination of network pharmacology prediction analysis and experimental validation. These results of the behavioral test showed that krill oil can improve memory deficits caused by METH exposure. Network pharmacology analysis demonstrated that krill oil may modulate function and apoptosis of neurons mainly *via* the regulation of the MAPK, cAMP, and dopaminergic synapse signaling pathway, which was verified by further experiment. Further experimental and clinical research trials of krill oil supplementation in METH abusers may help and support the identification of the potential therapeutic effects of krill oil on METH-induced cognitive and psychiatric disorders.

## Data Availability

The original contributions presented in the study are included in the article/Supplementary Material; further inquiries can be directed to the corresponding authors.
