# Supplementary figures and images for: Krill Oil Alleviated Methamphetamine-Induced Memory Impairment via the MAPK Signaling Pathway and Dopaminergic Synapse Pathway
Source: Front Pharmacol. 2021 Oct 29;12:756822. doi: 10.3389/fphar.2021.756822 (PMC8586701; doi:10.3389/fphar.2021.756822)

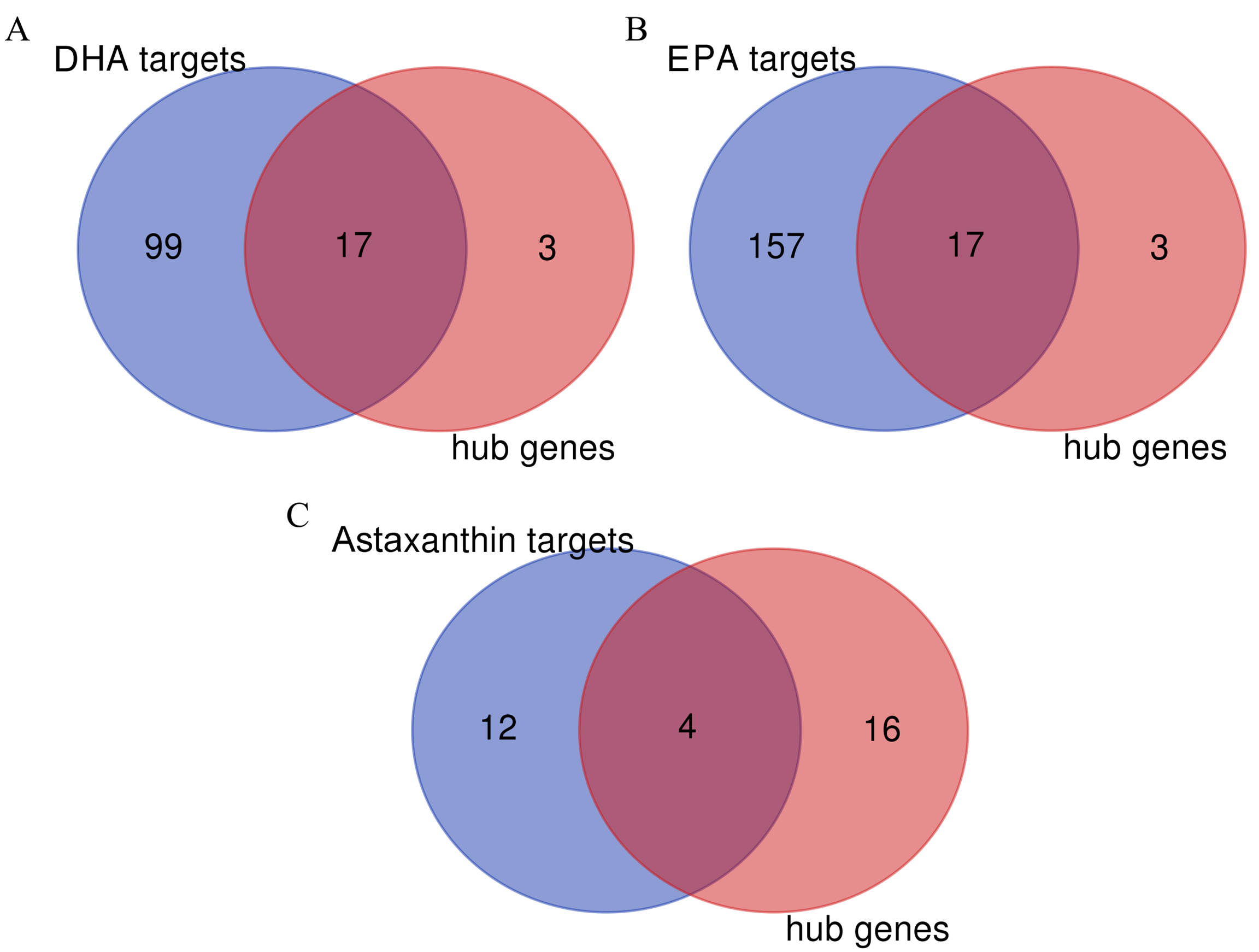

Supplement: Supplementary file 4 [file Image2.tif]

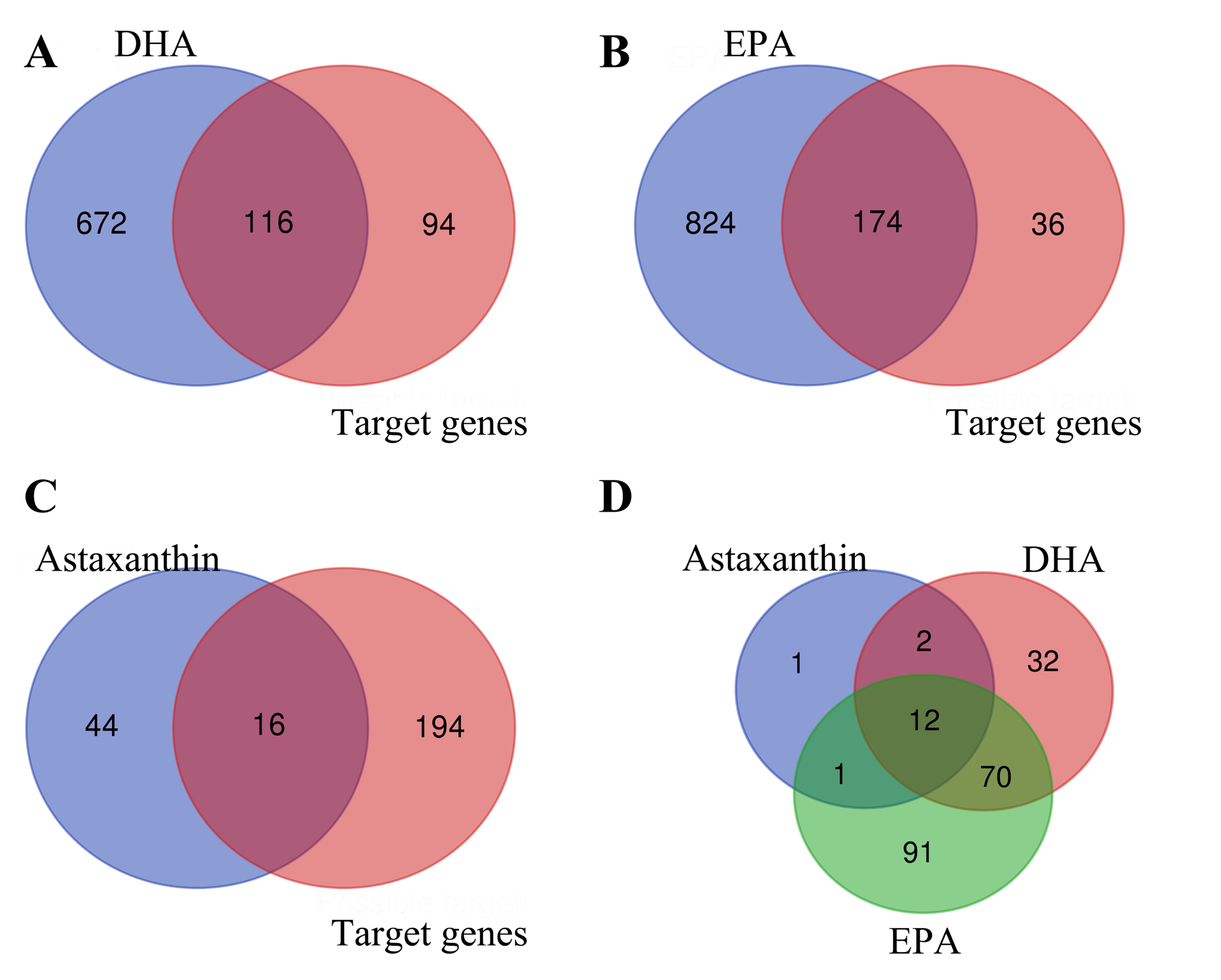

Supplement: Supplementary file 6 [file Image1.tif]
